# Diagnosis and prognosis prediction model for digestive system tumors based on immunologic gene sets

**DOI:** 10.3389/fonc.2023.1107532

**Published:** 2023-03-03

**Authors:** Lin Zhou, Chunyu Wang

**Affiliations:** ^1^ School of Information Science and Technology, University of Science and Technology of China, Hefei, Anhui, China; ^2^ School of Biological and Environmental Engineering, Chaohu University, Chaohu, Anhui, China

**Keywords:** digestive system tumors, immunologic gene set, diagnostic, prognostic, XGBoost

## Abstract

According to 2020 global cancer statistics, digestive system tumors (DST) are ranked first in both incidence and mortality. This study systematically investigated the immunologic gene set (IGS) to discover effective diagnostic and prognostic biomarkers. Gene set variation (GSVA) analysis was used to calculate enrichment scores for 4,872 IGSs in patients with digestive system tumors. Using the machine learning algorithm XGBoost to build a classifier that distinguishes between normal samples and cancer samples, it shows high specificity and sensitivity on both the validation set and the overall dataset (area under the receptor operating characteristic curve [AUC]: validation set = 0.993, overall dataset = 0.999). IGS-based digestive system tumor subtypes (IGTS) were constructed using a consistent clustering approach. A risk prediction model was developed using the Least Absolute Shrinkage and Selection Operator (LASSO) method. DST is divided into three subtypes: subtype 1 has the best prognosis, subtype 3 is the second, and subtype 2 is the worst. The prognosis model constructed using nine gene sets can effectively predict prognosis. Prognostic models were significantly associated with tumor mutational burden (TMB), tumor immune microenvironment (TIME), immune checkpoints, and somatic mutations. A composite nomogram was constructed based on the risk score and the patient’s clinical information, with a well-fitted calibration curve (AUC = 0.762). We further confirmed the reliability and validity of the diagnostic and prognostic models using other cohorts from the Gene Expression Omnibus database. We identified diagnostic and prognostic models based on IGS that provide a strong basis for early diagnosis and effective treatment of digestive system tumors.

## 1 Introduction

The digestive system consists of auxiliary organs of the digestive tract and gastrointestinal tract. Digestive system tumors have the highest mortality rate in the world. Digestive system tumors mainly include gastric cancer, colorectal cancer, esophageal cancer, etc., which come from different but related tissues and have their own unique clinical features, but also have some similar features (1). Common risk factors for gastrointestinal tumors include infection, smoking, alcohol consumption, high-fat diet, age, race, gender, family history, and geographic location (2). The therapeutic effect and survival time of tumors are closely related to the time of discovery, but there is still a lack of effective means for early detection, early diagnosis and early treatment of gastrointestinal tumors (3). Therefore, early diagnosis of gastrointestinal tumors, systematic research on the regulatory network during the development of gastrointestinal tumors, and development of new therapeutic strategies will be crucial to improving the survival rate of patients with gastrointestinal tumors, and are of great significance for improving the reduction of social pressure and disease burden (4–6). The current treatment methods, including surgery, radiation therapy, and immunotherapy, are constantly improving. In recent years, the research of immunotherapy has been steadily expanding, and research results have been continuously applied in clinical practice (7). However, due to the hidden early symptoms, the rapid development and aggression, the average survival time of patients with late DSC is still very low. Therefore, researchers are committed to discovering new features used for diagnosis or prognosis and improving treatment methods (8). There are already some very valuable studies, to assess the association of local expression of CD44 and CD24 with clinicopathologic features of disease in patients with large chronic kidney disease, the role of these markers as cerebrospinal fluid (CSF) was explored more fully (9). It has been found that Lg5High/DCLK1 high phenotype is significantly associated with the expression of early gastric cancer specimens, and its expression pattern can be considered a signature phenotype of gastrointestinal tumor subtypes (10).

The tumor immune microenvironment has been shown to play a key role in tumor development and influence clinical outcomes, and can serve as potential biomarkers to improve the reliability and accuracy of diagnosis and prognosis (11, 12). However, our understanding of its role remains incomplete due to the complexity and dynamics of the immune microenvironment (13). Tumor-infiltrating immune cells are part of a complex microenvironment (14). They play a key role in inhibiting or supporting tumor growth and development, can be effectively targeted by drugs, and are associated with patient survival (15). Gene expression profiling has become a mainstay of the TIME research field (16). However, due to its high heterogeneity and dynamics, studies on changes in individual genes cannot precisely dissect time. Typically, immune cell (IC) function is influenced by a group of related genes rather than a single gene. Therefore, the study of gene sets can provide new insights into cancer immunotherapy (17).

In this study, we evaluated the enrichment changes of IGS from ImmuneSigDB in patients with digestive system tumors. First, an IGS-based diagnostic model was established for tumor diagnosis, and then an IGS-based prognostic risk prediction model was established, its correlation with clinical and immune characteristics was evaluated, and a nomogram was constructed to make the results of the prediction model more readable It provides a powerful means for the early detection and prediction of DST.

## 2 Materials and methods

### 2.1 Raw data

The data used in this paper are from public databases. The DST cohort used to identify the immune gene set enrichment score consisted of 1345 patients in The Cancer Genome Atlas (TCGA). There are six main cancer types: ESCA, STAD, LIHC, PAAD, COAD, and READ. All transcriptome data and clinical data were downloaded from the TCGA database (https://tcga-data.nci.nih.gov/tcga/). Clinical data included clinical characteristics such as age, gender, survival time, survival status, and tumor status. Data was extracted from the TCGA database, strictly following TCGA-approved publication guidelines. Therefore, no ethics committee approval is required. The external validation dataset comes from the Gene Expression Omnibus (GEO) database.

### 2.2 Immunologic gene set and gene set variation analysis

ImmuneSigDB is a manually annotated database of approximately 5,000 gene sets in immunology from various cellular states, experimental manipulations, and genetic perturbations (18). ImmuneSigDB’s IGS (c7.ImmuneSigDB.v7.5) was obtained from the Molecular Signature Database (MSigDB). The enrichment score (ES) for each IGS in samples was calculated using the GSVA algorithm from the “GSVA” package in R. The GSVA enrichment algorithm is widely used in medical research (19–23).

### 2.3 Diagnostic analysis

Samples of primary or normal tissue were selected for further diagnostic analysis. First, the limma package was used for differential analysis, and the screened differential gene sets were used for subsequent diagnostic analysis. Patients were randomized into training and validation cohorts (4:1) using StratifiedKFold in scikit-learn. Extreme Gradient Boosting (XGBoost), is a scalable distributed gradient boosting decision tree machine learning library that provides parallel tree boosting capabilities and is an advanced machine learning library for regression, classification, and ranking problems (24). A diagnostic model was constructed on the training cohort using the XGBoost algorithm, and the sensitivity and specificity of the diagnostic model were analyzed by ROC curve. We searched for optimal parameters for XGBoost using Optuna (25).

### 2.4 Tumor subtypes based on immunologic gene set

According to the ES of IGSs, we used the consistent clustering method of the R package ConsensusClusterPlus (K-means, Euclidean distance, reps = 1000, pItem = 0.8, clusterAlg = “pam”, seed = 0) for the unbiased classification of all patients to explore the relationship between different tumor subtypes and patient prognosis (26). We used the square sum error in elbow (WSSE group; this method was to find the best cluster number by finding the “elbow point”) and the fastest falling point of the gap statistic (WK; the K value corresponding to the maximal value of gap) to evaluate the best class number K. In addition, we performed survival analysis for various immune subtypes.

### 2.5 Immune cell infiltration analysis

CIBERSORT (https://cibersortx.stanford.edu/) is a computational method for quantifying cellular components from gene expression profiles of ontology tissues (27). We used CIBERSORT to estimate the proportions of 22 ICs for digestive system tumors in TCGA and GEO. The immune and stromal scores were obtained by calculating the expression signatures of specific molecular biomarkers in immune and stromal cells using the ESTIMATE algorithm (https://r-forge.r-project.org) (28).

### 2.6 Prognostic analysis

For prognostic analysis, tumor samples with complete clinical characteristics and survival information were selected. Subsequently, eligible patients were randomized into training and validation cohorts (7:3) using R package caret. Predictive features were then screened from the training cohort using LASSO-Cox analysis. The coefficients characterize the risk score by using the R package glmnet according to the Least Absolute Shrinkage and Selection Operator (LASSO) algorithm. Optimal cut-off values for risk scores were calculated based on patient survival data using X-tile. Kaplan-Meier survival curves were used, and time-dependent ROC (survival ROC) curves were applied to assess the prognostic power of risk scores (29).

### 2.7 Validation of diagnostic and prognostic model using GEO dataset

Additional cohorts in the GEO database were used for the validation of the diagnostic and prognostic models according to the following inclusion criteria: (i) for the validation of the diagnostic model, the dataset provided tumor and normal samples containing mRNA expression levels in tissue samples; (ii) For validation of the prognostic model, the dataset provided patient survival information. Exclusion criteria were: (i) datasets with small sample sizes (n < 50); (ii) datasets using cell linesor animal samples. Therefore, we selected GSE37023, GSE23400, GSE37182, GSE90627, GSE22058, GSE62452 for diagnostic data, and GSE84433, GSE62452, GSE87211, GSE39582, GSE10186, GSE53624 for prognostic data to validate the results in the TCGA database.

### 2.8 Nomogram construction

The nomogram is based on multi-factor regression analysis, integrates multiple predictors, and then uses scaled line segments to draw on the same plane according to a certain proportion, and assigns each value of each influencing factor to each value ep26. Then, the individual scores are added to obtain the total score. Finally, the predicted value of the individual outcome event is calculated through the functional transformation relationship between the total score and the probability of occurrence of the outcome event. The total score projected on the bottom scale represents the probability of 2-year, 3-year, and 6-year overall survival. A calibration curve was drawn to compare expected and observed survival probabilities. The prognostic value of the nomogram and other clinical features was compared at 2, 3, and 6-year overall survival using ROC curves. The R package “rms” is used to draw nomograms and the R package ßurvivalROC” is used to draw ROC curves.

### 2.9 Statistical analysis

Statistical analysis was performed using R software (version 4.1.0). Continuous variables were expressed as mean ± standard deviation and compared using Student’s t-test or Wilcoxon rank-sum test. Categorical data were compared using the chi-square test. Use the python package xgboost to build diagnostic models. Least Absolute Shrinkage and Selection Operator (LASSO) regression models were performed using the “glmnet” and ßurvival” packages. Kaplan-Meier survival analysis with log-rank test was performed using the R package ßurvminer”. Differential expression analysis was performed using the “limma” package. Statistical significance was set at P < 0.05, shown as *P < 0.05, **P < 0.01, ***P < 0.001.

## 3 Results

### 3.1 Patient characteristics

**Figure 1:**
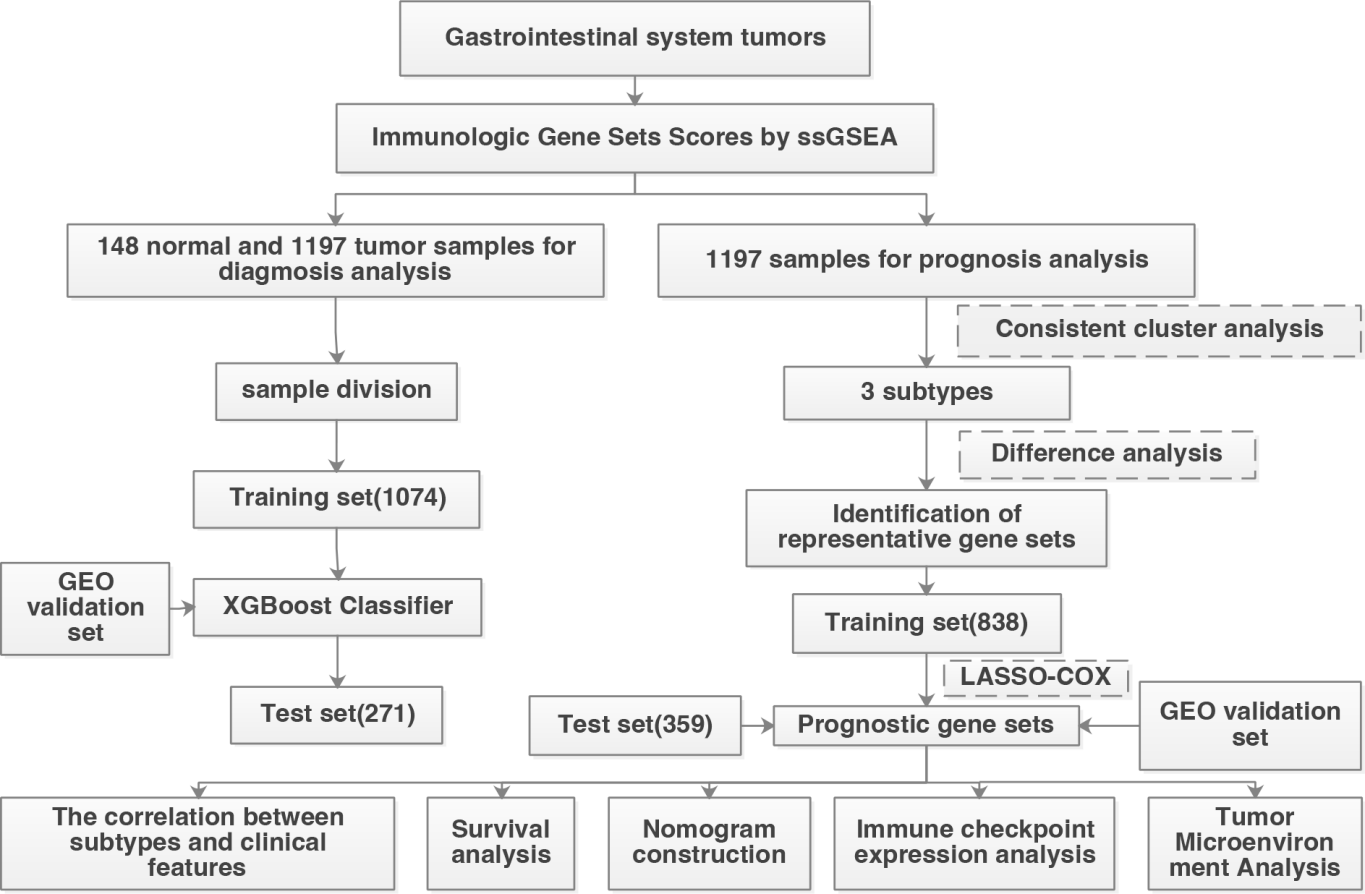
Workflow of this study.

According to the screening criteria, a total of 1345 patients were used for diagnostic analysis (including 148 normal samples and 1197 cancer samples) and 1197 tumor samples were used for prognostic analysis. The detailed distribution of the patients is summarized in **Table 1**, and the workflow of the study is illustrated in **Figure 1**.

**Table 1:** Distribution of all samples.

	Tumor sample (n=1197)	Percent (%)	Normal sample (n=148)	Percent(%)
Tumor type				
LIHC	276	23.1	50	33.8
STAD	263	22.1	32	21.6
COAD	303	25.3	41	27.7
READ	118	9.8	10	6.8
PAAD	117	9.7	4	2.7
ESCA	120	10	11	7.4
Diagnosis analysis				
Training	956	80	118	80
Validation	241	20	30	20
Prognosis analysis				
Training	838	70		
Validation	359	30		

### 3.2 Construction of diagnostic model based on immunologic gene set

**Figure 2:**
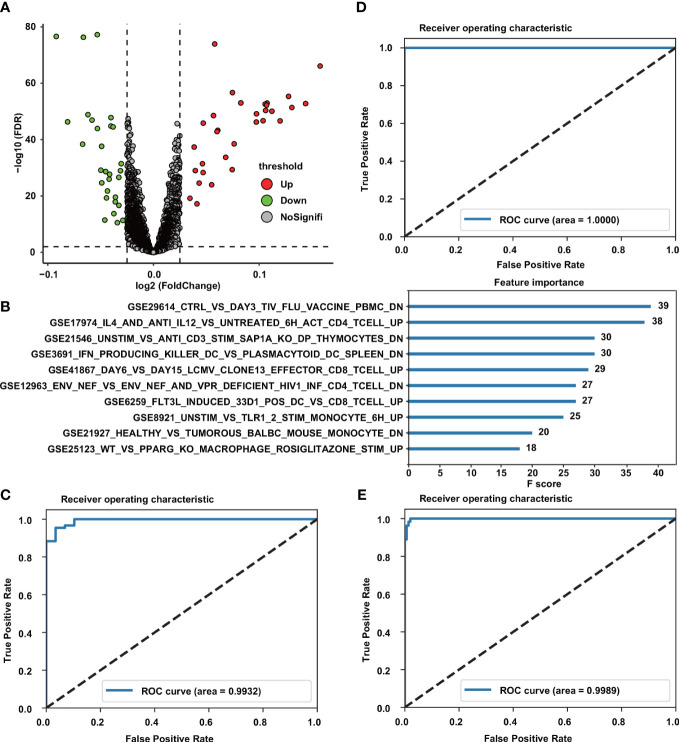
Construction of Diagnostic Model. **(A)** Volcano plot of differential analysis results between normal and cancer samples. Red, upregulated; green, downregulated. **(B)** Top10 feature importance in the diagnostic model. **(C–E)** ROC curves of the diagnostic model in the training cohort **(C)**, validation cohort **(D)** and the entire cohort **(E)**.

A total of 4,872 IGSs were obtained from ImmuneSigDB. IGSs for all 1345 digestive system tumors were calculated using the GSVA algorithm based on transcriptome RNA-seq data. Differential expression analysis showed that there were 60 significantly different gene sets (padj < 0.01) between normal samples and cancer samples, of which 31 gene sets were up-regulated and 29 were down-regulated (**Figure 2A**). All samples are then divided into training and validation sets while maintaining the same proportion of normal samples and cancer samples. A diagnostic model was constructed using the XGBoost algorithm based on the training set, and the ROC curve indicated that our model had high accuracy on the training set, validation set, and the entire dataset (AUCs of 1, 0.993, 0.999, respectively)(**Figures 2C–E**). Features importance analysis shows that GSE29614_CTRL_VS_DAY3_TIV_FLU_VACCINE_PBMC_DN and GSE17974_IL4_AND_ANTI_IL12_VS_UNTREATED_6H_ACT_CD4_TCELL_UP are the most important in the diagnostic model (**Figure 2B**).

### 3.3 Construction of tumor subtypes and prognostic model based on immunologic gene sets

**Figure 3:**
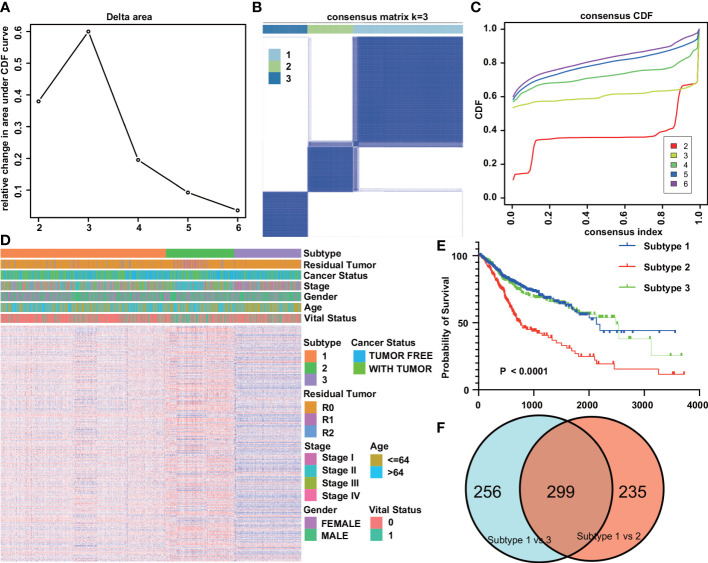
Tumor subtypes based on immunologic gene sets. **(A)** Delta area plot showed the relative change in area under the CDF curve. **(B)** Consensus matrices of the DST cohort for k=3. **(C)** Consensus cumulative distribution functions (CDF) of the consensus matrix for each k value (indicated by colors). **(D)** Gene set heatmap and clinicopathological features of the three subtypes identified. **(E)** Kaplan-Meier overall survival curves for the three subtypes. **(F)** Venn plot of the results of the analysis of differences between different subtypes.

Prognostic analysis of digestive system tumors used 1197 tumor samples. First, 1134 gene sets significantly associated with prognosis were screened from 4872 immune gene sets by univariate Cox regression analysis. Based on the prognosticIGS, the consistent clustering method of the R package “ConsensusClusterPlus” was used to classify the digestive system tumors into three subtypes (DSTS), namely subtype 1 (N=657), subtype 2 (N=273), and subtype 3 (N=267) (**Figures 3A-C**). The relationship between tumor type and subtype is shown in **Table S1**. The immune gene set enrichment score was the highest in subtype 2, followed by subtype 1, and the lowest in subtype 3 (**Figure 3D**). Kaplan-Meier survival analysis showed that subtype1 had the best prognosis, subtype 2 had the worst prognosis, and subtype 3 had an intermediate prognosis (**Figure 3E**). The above results suggest that DSTS can effectively discriminate patients with different prognosis.

**Figure 4:**
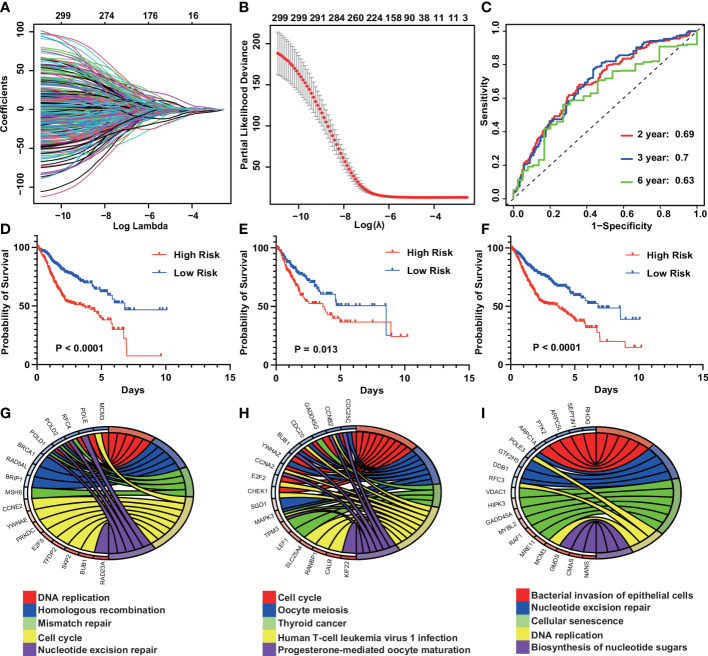
Construction of an IGS-based risk prediction model. **(A)** Least absolute shrinkage and selection operator (LASSO) coefficient profiles of the fractions of IGSs. **(B)** Ten fold cross-validation for tuning parameter selection in the LASSO model. **(C)** Risk score measured by survival receiver operating characteristic (ROC) curves in the training cohort. The area under the ROC curve (AUC) was 0.69, 0.7, and 0.63 at 2, 3, and 6 y, respectively. **(D–F)** Kaplan-Meier curves for overall survival by risk score group in the training **(D)**, validation **(E)** and entire cohorts **(F)**. **(G–I)** KEGG pathway analysis of the genes in Gene Sets 1–3.

To explore the mechanisms behind the prognostic differences between the different subtypes, we performed a differential analysis of the gene sets between the IGSS subtypes. There were 534 differential gene sets between subtypes 1 and 2, and 555 differential gene sets between subtypes 1 and 3. Then, taking the intersection of the two differential results, we obtained 299 differential gene sets (**Figure 3F**). These gene sets were then applied to LASSO regression analysis, and finally, a prognostic model consisting of nine gene sets was constructed (**Figures 4A, B**). The nine gene sets are: Gene Set 1 (GSE17301_ACD3_ACD28_VS_ACD3_ACD28_AND_IFNA2_STIM_CD8_TCELL_UP), Gene Set 2 (GSE20366EXVIVO_VS_HOMEOSTATIC_CONVERSION_TREG_DN), Gene Set3 (GE5542_IFNG_VS_IFNA_TREATED_EPITHEIAL_CELLS_24H_UP), Gene Set4 (GSE35543_IN_VIV

O_NTREG_VS_CONERTED_EX_ITREG_DN), Gene Set5 (GSE19198_1H_VS_24H_IL21_TREATED_TC

ELL_DN), Gne Set6 (GSE37301_AG2_KO_VS_RAG2_AND_ETS1_KO_NK_CELL_DN), Gene Set7 (GSE14699_DELETIONAL_TOLERANCE_VS_ACTIVATED_CD8_TCELL_DN), Gene Set8 (GSE17580_UNINFETED_VS_S_MANSONI_INF_TREG_UP), Gene Set9 (GSE6566_STRONG_VS_WE

AK_DC_STIMULATED_CD4_TCELL_UP). **Table S2** summarizes the genes included in gene sets 1-9. By calculating the sum of the products of ES and coefficients for each gene set, we can quantify the prognosis of each patient. Risk Score = (Gene Set 1 × -0.016) + (Gene Set 2 × -0.105) + (Gene Set 3 × -0.644) + (Gene Set 4 × 0.008) + (Gene Set 5 × 1.017) + (Gene Set 6 × -0.102) + (Gene Set 7 × -1.032) + (Gene Set 8 × 0.867) + (Gene Set 9 × -2.585). Use X-tile to calculate optimal cutoffs for risk scores based on patients’ survival data to classify patients in the training cohort into low-risk and high-risk groups. Kaplan-Meier curves were drawn to confirm that patients in the high-risk group had a significantly higher risk of survival in the training cohort (P < 0.0001) (**Figure 4D**). At the same time, Kaplan-Meier curves were also drawn in the validation and the whole cohort, consistent with the results of the training cohort, patients in the high-risk group had a lower overall survival time than those in the low-risk group (P = 0.013, P < 0.0001) (**Figures 4E, F**). Furthermore, the risk score showed the strong predictive power of 2-, 3-, and 6-year survival in the training cohort (AUC = 0.69, 0.7, and 0.63, respectively) (**Figure 4C**). Kyoto Encyclopedia of Genes and Genomes (KEGG) pathway analysis showed that gene sets 1-9 most enriched in DNA replication, Cell cycle, Bacterial invasion of epithelial cells, Mannose type O-glycan biosynthesis, Viral myocarditis, Fatty acid degradation, Human T-cell leukemia virus 1 infection, Hematopoietic cell lineage, Aldosterone synthesis and secretion (**Figures 4G-I**). The enrichment of the other six gene sets is shown in **Figure S1**.

### 3.4 Correlation of risk prediction model with immune cell infiltration and expression of immune checkpoints

**Figure 5:**
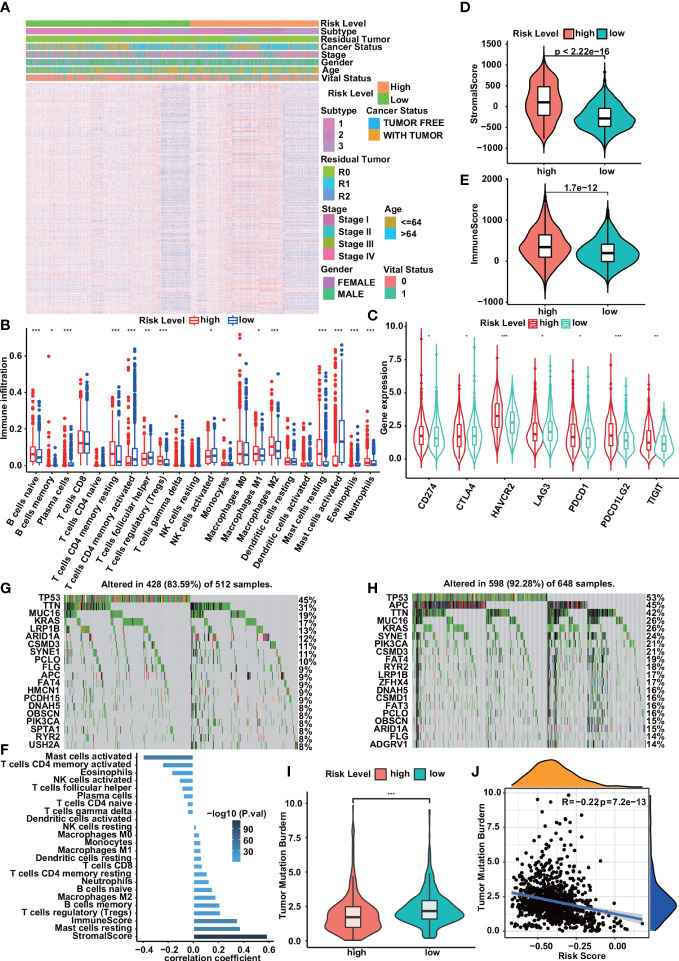
Correlation of risk prediction model with immune cell infiltration,immune checkpoints, and somatic mutation. **(A)** Heatmap of immune gene set enrichment scores for high- and low-risk groups. **(B)** Immune cell infiltration in low- and high-risk groups. **(C)** Compared with the low-risk group, the expression levels of immune checkpoint molecules in the high-risk group were significantly increased. **(D, E)** Violin plots show significant associations between risk group and stromal score **(D)**, immune score **(E)**. **(F)** Correlation between risk score and infiltrating immune cell density and stromal/immune score. **(G, H)** Somatic mutation profiles of the 20 most frequently mutated genes in low- and high-risk groups. **(I, J)** Correlations of risk scores with TMB. The violin plot showed that the low-risk group had higher TMB than the high-risk group **(I)**. TMB was significantly negatively correlated with risk score **(J)** . Statistical significance was set at P<0.05, shown as *P<0.05,**P<0.01, ***P<0.001.

The heatmap of the high- and low-risk groups shows that the high-risk group has a higher gene set enrichment score (**Figure 5A**). Infiltration of 22 ICs in the digestive system cohort was analyzed using the CIBERSORT package. We found that the infiltration of B cells memory, Plasma cells, T cells CD4 memory activated, T cells follicular helper, Mast cells activated, and Eosinophils was higher in the low-risk group. In contrast, B cells naïve, T cells CD4 memory resting, T cells regulatory (Tregs), Neutrophils, and Mast cells resting had higher infiltration levels in the high-risk group (**Figure 5B**). The stromal score and immune score of all samples were obtained using the ESTIMATE algorithm, and the scores ranged from -1009.408 to 1112.625 and -607.9489 to 1571.0439, respectively. There were significant differences in the stromal score and immune score in the high and low-risk groups, and they were all higher in the high-risk group (**Figures 5D, E**). Correlation analysis showed that risk scores were negatively correlated with Mast cells activated, T cells CD4 memory activated, and Eosinophils, while positively correlated with stromal scores, immune scores, Mast cells resting, and T cells regulatory (Tregs) (**Figure 5F**). In addition, we compared the expression of immune checkpoint molecules including CD274, PDCD1, PDCD1LG2, CTLA4, HAVCR2, LAG3, and TIGIT in high-risk and low-risk groups. We found that the expression levels of immune checkpoint molecules were significantly higher in the high-risk group compared with the low-risk group (**Figure 5C**). We analyzed simple nucleotide variation data from the digestive system cohort to characterize somatic mutations in high- and low-risk groups. We found that the overall mutation rate was significantly higher in the low-risk group (92.28 *vs*. 83.59). Except for TP53, KRAS, TTN, MUC16, LRP1B, ARID1A, CSMD3, FLG, SYNE1, APC, PIK3CA, RYR2, OBSCN, PCLO, FAT4, and DNAH5, these genes had high mutation rates in both high-risk and low-risk groups. Compared with the low-risk group, the high-risk group had higher mutation rates of HMCN1, PCDH15, SPTA1, and USH2A, while compared with the high-risk group, the low-risk group had higher mutation rates of CSMD1, ZFHX4, FAT3, ADGRV1mutation rate is higher (**Figures 5G, H**). In addition, the risk score was also significantly negatively correlated with TMB and had higher values in the low-risk group (**Figures 5I, J**).

### 3.5 Nomogram construction

**Figure 6:**
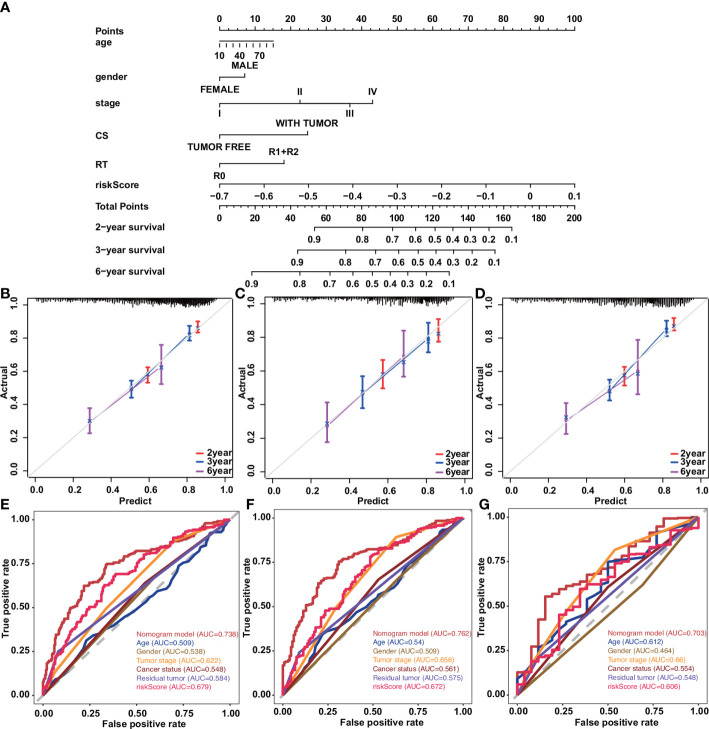
Construction and validation of a nomogram in patients with digestive system tumors. **(A)** Nomograms used to predict 2-, 3-, and 6-year overall survival for patients in the training cohort. **(B–D)** Calibration curves of nomograms in terms of agreement between predicted and observed 2-, 3-, and 6-y outcomes in the training **(B)**, validation **(C)**, and entire **(D)** cohorts. Dashed line at 45°represents perfect prediction, and the actual performance of our nomogram is the red, blue, and pink lines. **(E)** ROC curve for predicting 2-year OS by risk score. **(F)** ROC curve for predicting 3-year OS by risk score. **(G)** ROC curve for predicting 6-year OS by risk score.

The nomogram transforms the complex regression equation into a visual graph, making the results of the prediction model more readable and facilitating the evaluation of patients. Construct a prognostic nomogram based on clinical information such as age, tumor stage, and cancer status, and generate a quantitative method for predicting the prognosis of patients with cancer of the digestive system (**Figure 6A**). Calibration curves for nomograms showed good agreement between predictions and observations in the training cohort. A good agreement was also observed across validation and the entire cohort (**Figures 6B–D**). Moreover, the 2-year, 3-year, and 6-year ROC curves directly show the value of risk factors. The nomogram had the highest accuracy, with areas under the ROC curve (AUC) of 0.738, 0.762, and 0.703, indicating appropriate clinical applicability of the nomogram (**Figures 6E–G**).

### 3.6 Use GEO datasets to verify diagnostic model and prognostic model

**Figure 7:**
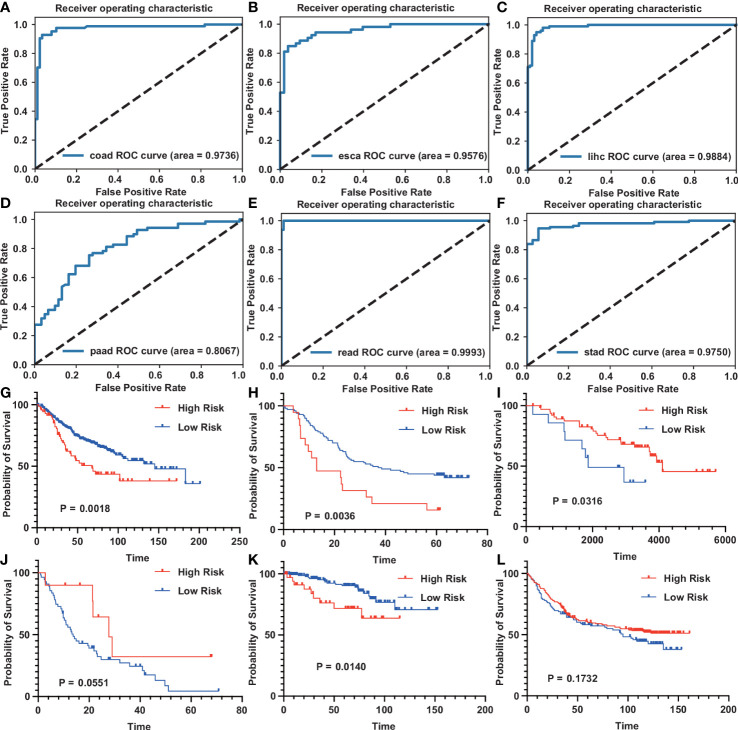
GEO datasets verification diagnostic model and prognostic model for digestive system tumors **(A–F)** The ROC curve of the diagnostic model on the GEO dataset. [**(A)**, COAD; **(B)**, ESCA; **(C)**, LIHC; **(D)**, PAAD; **(E)**, READ; **(F)**, STAD]. **(G–L)** The survival curve of the prognostic model on the GEO dataset. [**(G)**, COAD; **(H)**, ESCA; **(I)**, LIHC; **(J)**, PAAD; **(K)**, READ; **(L)**, STAD].

Get GSE37182 (COAD), GSE23400 (ESCA), GSE22058 (LIHC), GSE62452 (PAAD), GSE90627 (READ), GSE37023 (STAD) from the GEO database to verify the diagnostic model. We used these datasets to evaluate the ability of tumors and normal tissues in diagnostic models, showing the high accuracy of diagnosis (AUC was 0.9736, 0.9576, 0.9884, 0.8067, 0.9993, 0.975) (**Figures 7A-F**). Use GSE39582 (COAD), GSE53624 (ESCA), GSE10186 (LIHC), GSE62452 (PAAD), GSE87211 (READ), and GSE84433 (STAD). Among them, COAD, ESCA, and READ are consistent with our TCGA database. Higher risk scores indicate that patients are more likely to survive. However, the survival rate of patients with high-risk scores in LIHC, PAAD, and STAD is higher (**Figures 7G-L**).

## 4 Discussion

It is worth noting that the latest development of new cancer treatment methods is mainly concentrated on early intervention. Munoz and Plevritis et al. (30) propose a predictive model that uses estrogen receptors and human epidermal growth factor receptors 2 to determine the potential survival results. Similarly, Chen et al. (31) use lncRNA data in the TCGA database to obtain five lncRNA signatures for independent risk factors for OC recurrence. HUANG et al. (32) use clinical pathological risk factors to build a radiological characteristics and radioactive group diagram of lymph nodes metastasis of colorectal cancer, which facilitates the preparation prediction before surgery. However, most of these studies are based on the analysis of single genes. In our research, we focus on the collection of immune genes, not a single gene, which will improve our understanding of the overall function of IC (33–35).

First, we used the XGBoost algorithm to construct a diagnostic model based on a set of 57 immune genes differentially expressed between normal and cancer samples. The high AUC values indicate that our model is accurate and effective in diagnosing tumors in the digestive system, and that the immune system is involved in the development and progression of cancer.

There were 534 and 555 differentially expressed immune gene sets between subtypes 1 and 2 and between subtypes 1 and 3, respectively. Although there were also significant differences in the expression of IGS between subtypes 2 and 3 (n = 486), we found poor prognosis for both subtypes. Subtype 1 had the best prognosis compared to subtypes 2 and 3. There are also different clinical, molecular and immune associations with subtypes 2 and 3. Therefore, we compared the differential expression of IGS between subtypes 1 and 2 and between subtypes 1 and 3 to better elucidate the underlying mechanisms of subtype 1.

Discovery of nine gene sets to construct IGS-based prognostic models provides new insights into functional diversity of TIME, leading to potential biomarkers and therapeutic targets for cancer management. The Kaplan-Meier curve confirmed that patients with high-risk scores had a higher chance of survival in the training cohort. The results of the internal and external validation sets were largely consistent with the above results.

To improve prognostic accuracy, we combined risk scores, age, sex, tumor stage, cancer status, and residual tumors to construct a lineup and ROC curves for 2, 3, and 6 years of survival. The results show that the line diagram has good clinical applicability. In addition, calibration curves show that prognostic immune scores predict clinical outcomes in patients. Taken together, this study provides a comprehensive immune map of tumors in the digestive system, resulting in diagnostic and prognostic models that can be used as biomarkers for early diagnosis to initiate treatment and predict patient survival.

Numerous studies have reported the influence of tumor microenvironment on tumor development and prognosis including esophagus (36), pancreas (37), colorectal cancer (38), gastric cancer (39) and melanoma (40). However, this study still has some limitations. First, the patients in the TCGA database that we used lacked some clinical information, such as acute infection or immune system disease, which would affect the results of the analysis. In addition, information on more meaningful risk factors for diagnosis and prognosis, such as smoking, alcohol consumption, and family history, was incomplete. In the future, we need to collect more complete clinical information for analysis to further improve the reliability of the results. Second, because all samples were from retrospective collections, further prospective studies are needed to validate the results. We will apply the analytical results to the clinic.

## 5 Conclusions

All in all, we have established an IGS-based diagnostic model that enables accurate early diagnosis of digestive system tumors. In addition, we construct DSTS to provide new insights into the relationship between immune processes and TIME features, while IGS-based prognostic prediction models can accurately predict the prognosis of DST patients, and their predictive ability is verified in GEO data. Diagnostic and prognostic models can be used as useful tools for early diagnosis of biomarkers and the development of new strategies for cancer immunotherapy.

## Data availability statement

The original contributions presented in the study are included in the article/**Supplementary Material**. Further inquiries can be directed to the corresponding author.

## Author contributions

LZ and CW conceived and designed the study interpreted the results and wrote the manuscript. LZ and CW collected data and helped interpret the data. All authors contributed to the article and approved the submitted version.
