# Supplementary figures and images for: Diagnosis and prognosis prediction model for digestive system tumors based on immunologic gene sets
Source: Front Oncol. 2023 Mar 3;13:1107532. doi: 10.3389/fonc.2023.1107532 (PMC10020235; doi:10.3389/fonc.2023.1107532)

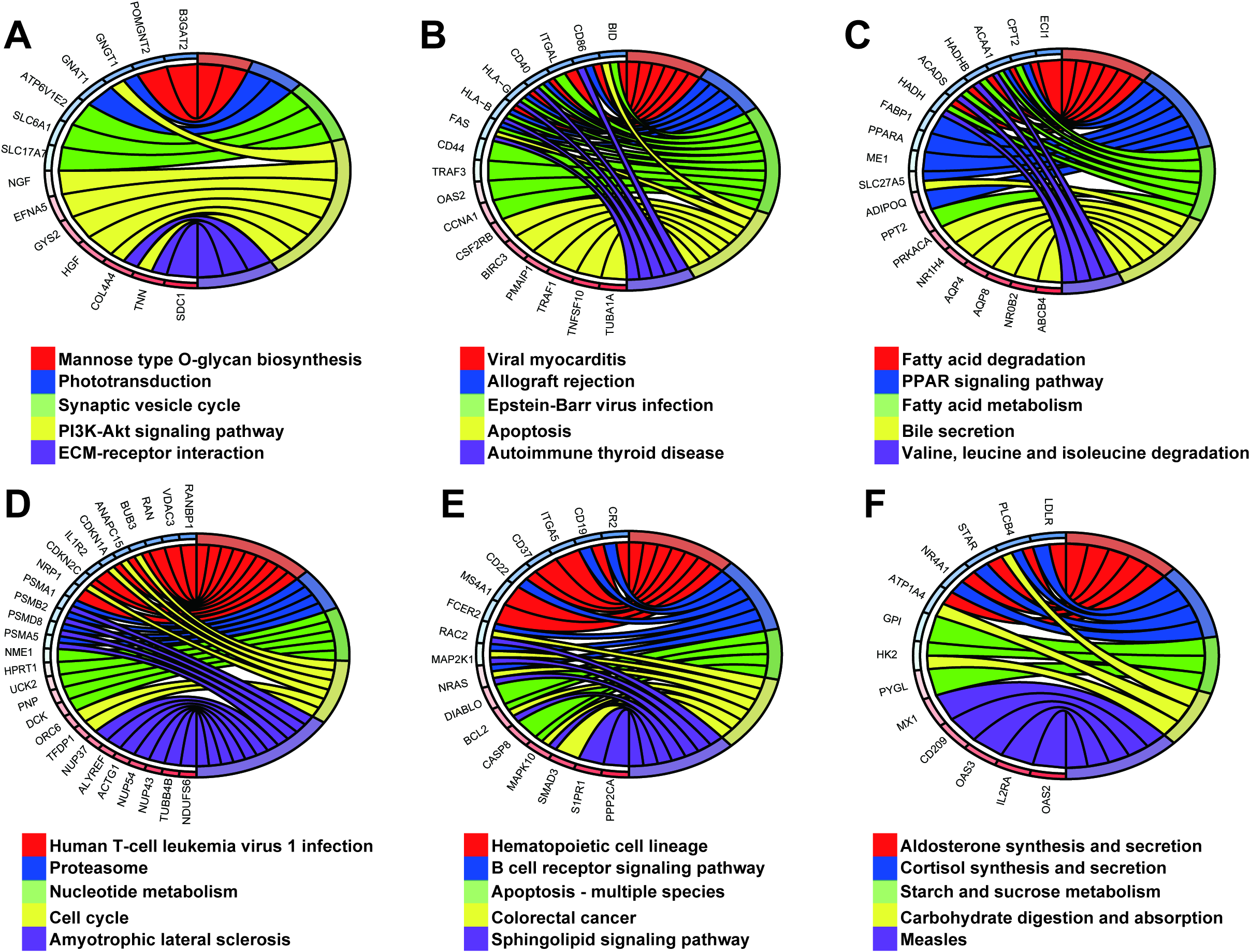

Supplement: Supplementary file 1 [file Image_1.tif]
